# A microplate technique to simultaneously assay calcium accumulation in endoplasmic reticulum and SERCA release of inorganic phosphate

**DOI:** 10.1186/1480-9222-14-4

**Published:** 2012-04-02

**Authors:** David C McMullen, William S Kean, Ajay Verma, Jeffrey T Cole, William D Watson

**Affiliations:** 1 Department of Neurology, Uniformed Services University of the Health Sciences, B-3059, 4301 Jones Bridge Road, Bethesda, MD 20814, USA; 2 Department of Neurology, Uniformed Services University of the Health Sciences, B-3009, 4301 Jones Bridge Road, Bethesda, MD 20814, USA; 3 Department of Neurology, Uniformed Services University of the Health Sciences, B-3012, 4301 Jones Bridge Road, Bethesda, MD 20814, USA

**Keywords:** Calcium, SERCA activity, Microsomes, Inorganic phosphate, Malachite green

## Abstract

Traditional analyses of calcium homeostasis have separately quantified either calcium accumulation or release mechanisms. To define the system as a whole, however, requires multiple experimental techniques to examine both accumulation and release. Here we describe a technique that couples the simultaneous quantification of radio-labeled calcium accumulation in endoplasmic reticulum (ER) microsomes with the release of inorganic phosphate (Pi) by the hydrolytic activity of sarco-endoplasmic reticulum calcium ATPase (SERCA) all in the convenience of a 96-well format.

## Introduction

Precise calcium (Ca^2+^) regulation is essential to most cellular functions and cell survival, while Ca^2+ ^dystasis can lead to cell death [1]. Eukaryotic cells regulate intracellular Ca^2+ ^concentration and distribution by transport across membranes into organelles or the extracellular environment using a complex system of ion pumps, exchangers, channels, and binding proteins [2,3]. Both the extracellular and total cellular Ca^2+ ^concentration is typically 2 mM, while the free concentration in the cell cytosol at rest is maintained at 100 nM - four orders of magnitude lower than the extracellular concentration [4]. This high electrochemical gradient makes Ca^2+ ^an ideal second messenger, with small local cytosolic changes in concentration representing large fractional changes. The endoplasmic reticulum (ER) is a major intracellular store of second messenger Ca^2+^, and this laboratory established a technique to quantify ^45^Ca^2+ ^accumulation in ER microsomes and identified a novel Ca^2+ ^pool in the central nervous system [5,6]. Ca^2+ ^accumulates in the ER is via the ubiquitously expressed magnesium, ATP-dependent sarco-endoplasmic reticulum calcium ATPases (SERCAs) which rapidly transport excess Ca^2+ ^from the cytosol into the ER lumen [7,8]. There are three genes that encode SERCAs in the mammalian genome and tissue specific alternative splicing of these gene products results in at least 11 known isoforms [9-11]. Of these, SERCA2b is expressed almost ubuiquitously whereas the others demonstrate temporal and tissue specific expression. In addition, all known isoforms are inhibited by the general P-type ATPase inhibitors such as La^3+ ^and orthovanadate, as well as the more potent and specific inhibitor thapsigargin (TG) [12,13].

Via conformational changes, SERCAs transfer two Ca^2+ ^ions from the cytoplasm into the ER lumen per molecule of ATP hydrolyzed [9,14]. During this process, SERCAs transiently form a covalent bond with the gamma phosphate group of ATP [15]. Following the transport of Ca^2+ ^ions into the lumen, phosphate is released as inorganic phosphate (P*i*). Experimentally, SERCA activity can be measured using ATP-γ-^32^P in radioassays. A radiolabeled phosphorylated intermediate (E-P) of SERCA can be isolated in microsomal vesicles under appropriate conditions [16]. This E-P intermediate is strongly inhibited by TG, a potent and irreversible SERCA inhibitor, and partially by 2,5-di- (-butyl) benzohydroquinone [6]. Alternatively, SERCA activity can be measured by quantifying the radiolabeled inorganic phosphate produced from ATP-γ-^32^P hydrolysis.

The standard trace ^45^Ca^2+ ^accumulation assay measures the net luminal influx of Ca^2+^. Experimentally, the net amount of ^45^Ca^2+ ^that accumulates over time is due to SERCA-dependent uptake counterbalanced by loss of Ca^2+ ^via passive leak pathways and microsomal resident release channels (ie, inositol-triphosphate receptors, IP^3^Rs, and ryanodine receptors, RyRs). Thus net measurement of ^45^Ca^2+ ^accumulation alone as a single measure is insufficient to quantify SERCA activity. By coupling the radioactive ^45^Ca^2+ ^accumulation assay in microsomes with quantification of ATP hydrolysis and P*i *release into the post-assay eluate, SERCA-dependent Ca^2+ ^accumulation in comparison to SERCA activity (ATP hydrolysis) can simultaneously be quantified. This coupled approach using a combination of radioisotopic and colorimetric assays provides a new, more powerful assay to investigate ER Ca^2+ ^flux and can be highly useful for understanding the mechanism of drug, toxin and ATP action on ER Ca^2+ ^regulation.

## Materials and methods

Male Sprague Dawley rats were purchased Taconic farms (Derwood, MD), ^45^Ca^2+ ^was purchased from MP biomedicals (Solon, OH) and all other chemicals were purchased from Sigma-Aldrich chemical company (St. Louis, MO). For a complete step by step procedure, please see Additional file 1.

### Tissue preparation

All animal procedures were approved by the Uniformed Services University of the Health Sciences Institution for Animal Care and Use Committee (IACUC) in accordance with international guidelines on the ethical use of animals. Whole brain microsomes were prepared as described previously [17]. Briefly, male Sprague-Dawley rats were anaesthetized with CO^2 ^and decapitated. The entire brain was quickly removed and minced on an ice-cold glass plate containing homogenization buffer [20 mM HEPES, pH adjusted to 7.35 with KOH, 0.25 M sucrose, 100 uM ethylenediaminetetraacetic acid (EDTA) and 1x protease inhibitor cocktail (Sigma)], then homogenized in 10 volumes (v/v) of ice cold buffer using a motor-driven glass-teflon homogenizer. After centrifuging the chilled homogenate at 10,000 × *g *for 15 minutes at 4°C, the resulting pellet containing nuclei, mitochondria and cellular debris was discarded. The supernatant was retained for subsequent ultracentrifugation at 100,000 × *g *for one hour at 4°C. The resultant supernatant was carefully decanted and discarded, and the pellet retained and washed twice with incomplete ice cold homogenization buffer (minus EDTA or protease inhibitors). The washed pellet was re-suspended in incomplete ice cold homogenization buffer and the protein concentration determined using the BCA method (Pierce, Rockford, IL). Microsomes were diluted with the same buffer to a final protein concentration of 2.5 mg/ml and stored at -80°C in 1.2 mL aliquots.

### ^45^Ca^2+ ^assay

This assay was developed in our lab to run in a 96-well microplate format, using a 0.45 μm glass fiber type B MAFB micro filter plate from Millipore (Billerica, MA) to allow data collection from both the microsomes and the filtrate. The uptake reaction was run in assay buffer prepared to our specifications by World Precision Instruments (Sarasota, Fl) that contained 20 mM HEPES, pH adjusted to 7.35 with KOH, 80 mM potassium chloride (KCl), 3% (w/v) polyethylene glycol (PEG, average molecular weight of 10,000), 5 mM sodium azide (served both as an anti-microbial agent and an inhibitor of mitochondrial activity), 25 mM potassium oxalate, and 200 μM CaCl_2 _that was EGTA-chelated down to desired concentrations of free Ca^2+^. During assay development, it was found experimentally that a large, non-reactive bioreagent was required to maintain osmolar stability for assay function, for SR/ER preparations to reliably uptake as well as release Ca^2+ ^over time in the presence of known classical second messengers. Without appropriate addition of polyethylene glycol (PEG), adding organelles with lumens, like ER, potentially burst from osmolar influx, even without activated Ca^2+ ^uptake additions (ATP/Mg^2+^/Ca^2+ ^for SERCA activity). In presence of uptake additions, highly variable, unpredictable ^45^Ca^2+ ^accumulation occurs over time due to influx of osmolar gradients competing with Ca^2+^. Unless otherwise specified, all experiments were conducted in a buffer with 300 nM free Ca^2+^. Free Ca^2+ ^concentrations were verified by a Ca2+ - EGTA calculator program [18]. In our case, the assay buffers were prepared in 20 L lots with an initial Ca^2+ ^concentration of 200 μM and required 361.6 mL of 10 mM EGTA to titrate the free Ca^2+ ^concentration to the desired concentration of 300 nM. Prior to beginning the experiment, additional fresh reagents were prepared and added to 1.2 mL of assay buffer. This pre-mix includes: 2 mM adenosine 5' triphosphate (ATP), 2 mM MgCl_2_, 1,4 dithiothreitol (DTT), 5 mM phosphocreatine (PCr), and 20 U/mL creatine phosphokinase (CPK). SERCA activity is Mg^2+^- and ATP-dependent, and for every two Ca^2+ ^ions transported, hydrolyzes one ATP to ADP. The CPK and PCr serve as a buffer ATP-regeneration system replenishing ATP stores to maintain SERCA activity under experimental conditions [19,20]. Radiotracer ^45^Ca^2+ ^was lastly added to the assay buffer at a final concentration of 0.2 μCi/mL.

To start the experiment, a 96-well plate was loaded with assay buffer (added to bring total volume to 250 μL), microsomes (100 μg/mL), test reagents (e.g., TG), and the pre-mix. The covered microplate was incubated at 37°C for 60 minutes, and the reaction terminated by filtration using a Millipore vacuum plate base (MAVM0960R). We previously determined the rate of accumulation was still in the linear phase at 60 minutes. We measured microsomal Ca^2+ ^accumulation for a total of 180 minutes in 30 minute intervals and did not see a decrease in the reaction slope until after 90 minutes (data not shown). The plastic filtrate capture basin normally used was replaced by a clear microplate placed in the vacuum plate base immediately beneath the filter plate such that all cells were aligned to capture the filtrate. Because of their size and charge, microsomes with accumulated luminal ^45^Ca^2+ ^remained trapped in the microplate wells [17]. Filters were washed twice with ice-cold wash buffer containing 10 mM HEPES-KOH (pH 7.3), 100 mM KCl, 3% (w/v) PEG, 10 mM potassium oxalate, 5 mM MgCl_2_, and 2 mM EGTA to remove non-specific signal. Finally, 50 μL of Ready Value scintillation fluid (Beckman, Brea, CA) was added to each well of the filter plate and the radioactive signal was measured in a Wallac microbeta liquid scintillation counter (Perkin Elmer, Shelton, CT) to determine ^45^Ca^2+ ^accumulation. Non-specific uptake was considered ^45^Ca^2+ ^accumulation in the presence of 10 μM A23187, a potent and highly selective Ca^2+ ^ionophore.

### SERCA activity

SERCA activity was measured using a colorimetric assay that quantifies the amount of P*i *that complexes with ammonium molybdate and malachite green following release from SERCA-mediated ATP hydrolysis [21]. All glassware and filter paper used in the subsequent steps were previously rinsed with 4 M HCl, and all solutions were prepared with ultra-pure water to reduce background P*i *contamination. Briefly, the reagent to quantify P*i *was prepared by mixing 1 volume of 10% (w/v) ammonium molybdate in 4 M HCl with 3 volumes 0.2% (w/v) malachite green in 4 M HCl, followed by stirring for 30 min then gravity filtration. The initial 20% of the filtrate was discarded due to dilution by the HCL rinse. Inorganic phosphate reagent was stored in the dark for no more than two weeks before replacement. Experiments were conducted to optimize parameters such as time, enzyme concentration, and reagent concentrations,(data not shown). When P*i *is complexed with ammonium molybdate and malachite green in 4 M HCl, it creates a green color which can be quantified by reading the absorbance spectrophotometrically at 660 nm in a FLUOstar Omega (BMG Labtech, Cary, NC) and compared to a standard curve of known P*i *concentrations [22].

A standard curve was generated by preparing a stock of 10 mM NaHPO^4 ^in assay buffer which was stored at -20°C when not in use. This stock solution was then further diluted in assay buffer to generate a standard curve ranging from 0 to 15 nmols of PO_4_^2-^, each in a final volume of 200 μL. Fifty μL of malachite green/ammonium molybdate dye reagent was added to each sample and color allowed to develop for 10 minutes at room temperature before being quantified by absorbance spectrophotometric analysis at 660 nm in a FLUOstar Omega (BMG Labtech, Cary, NC). To quantify P*i *in the filtrate, a 15 μL aliquot was removed and combined in a fresh microplate containing 185 μL assay buffer and 50 μL dye reagent. Once again, the colorimetric reaction was allowed to proceed for 10 minutes before the OD_660 _was measured spectrophotometrically.

## Results

**Figure 1:**
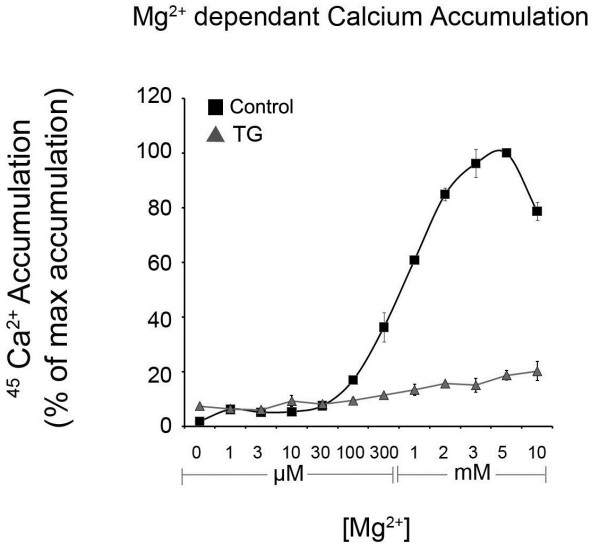
**Mg^2+ ^concentration dependence of ^45^Ca^2+ ^uptake in ER microsomes isolated from male rat brain**. Net ^45^Ca^2+ ^accumulation was measured by liquid scintillation counting after the uptake reaction was allowed to proceed for 60 minutes at 37°C in buffer containing 300 nM free Ca^2+ ^and 2 mM ATP (see materials and methods). Data are means ± SEM, n = 3.

These experiments establish a more robust technique coupling quantification of Ca^2+ ^radiotracer accumulation in ER microsomes with the hydrolysis and release of P*i *by ER resident Ca^2+^-ATPases (SERCAs). SERCA activity is highly dependent on the availability of both Mg^2+ ^and ATP [23]. Figure 1 depicts the dependence of ^45^Ca^2+ ^accumulation in microsomes on increasing concentrations of Mg^2+^. In this experiment, ATP concentration was held constant at 2 mM, while Mg^2+ ^concentration was increased from 0 to 10 mM. Calcium accumulation was measured without (control) or with 1 μM TG. A clear dependence on Mg^2+ ^was observed with activity not rising above background levels until 100 μM of Mg^2+ ^was present. A plateau in activity was obtained when Mg^2+ ^levels reached 3-5 mM. As expected, Mg^2+ ^concentrations above 5 mM competitively inhibited Ca^2+ ^binding sites on the SERCA, thus reducing Ca^2+ ^accumulation in the microsomes [17,24]. Also, as reported previously, 1 μM TG potently inhibited ^45^Ca^2+ ^accumulation [6,25]. Having determined the optimal concentration of Mg^2+ ^to maximize Ca^2+ ^accumulation, 3 mM Mg^2+ ^was used for all subsequent experiments.

**Figure 2:**
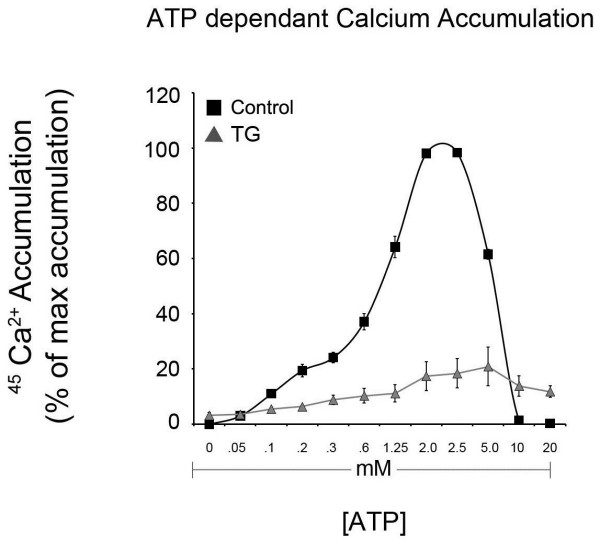
**ATP concentration dependence of ^45^Ca^2+ ^uptake in ER microsomes isolated from male rat brain**. Net ^45^Ca^2+ ^accumulation was measured by liquid scintillation counting after the uptake reaction was allowed to proceed for 60 minutes at 37°C in buffer containing 300 nM free Ca^2+ ^and 3 mM Mg^2+ ^(see materials and methods). Data are means ± SEM, n = 3.

To determine the best ATP concentrations (Figure 2), Ca^2+ ^accumulation assays were repeated using a constant concentration of Mg^2+ ^(3 mM). As with the low levels of Mg^2+ ^in Figure 1, Ca^2+ ^accumulation was not detectable at ATP concentrations below 50 μM. V_Max _was reached at 2.5 mM ATP. Higher concentrations of ATP stimulate the opening of IP_3 _release channels thereby reducing Ca^2+ ^accumulation in ER microsomes [16]. In addition, roughly 80% of the ATP-dependent ^45^Ca^2+ ^accumulation was TG-sensitive.

**Figure 3:**
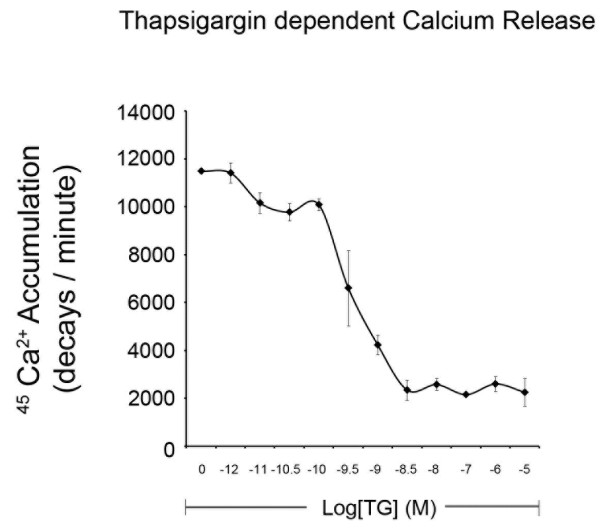
**Inhibition of Mg^2+ ^and ATP-dependent ^45^Ca^2+ ^accumulation by TG in ER microsomes isolated from male rat brain**. The ATP concentration was 2 mM, Mg^2+ ^concentration was 3 mM and the free Ca^2+ ^concentration was 300 nM. Data are means ± SEM, n = 3.

Having optimized the Mg^2+ ^concentration, and chosen a physiologically relevant ATP level (2.0 mM), we investigated the pharmacokinetics of SERCA inhibition on ER Ca^2+ ^accumulation and P*i *release under the same assay conditions, with data collected simultaneously. TG, experimentally the most potent and commonly used specific inhibitor of SERCA [25,26], significantly reduced Ca^2+ ^accumulation in ER microsomes (Figure 3). At low concentrations of TG (100 pM to 50 nM) there was a rapid, significant reduction in ER Ca^2+ ^accumulation, representing a TG-sensitive Ca^2+ ^pool (TG-S) [6]. Above these concentrations, however, there is no further reduction of Ca^2+ ^accumulation until doses in excess of 100 μM are reached, representing the TG-resistant (TG-R) pool (data not shown). The TG-R pool represented approximately 20% of the total sequestered Ca^2+ ^in our assays.

**Figure 4:**
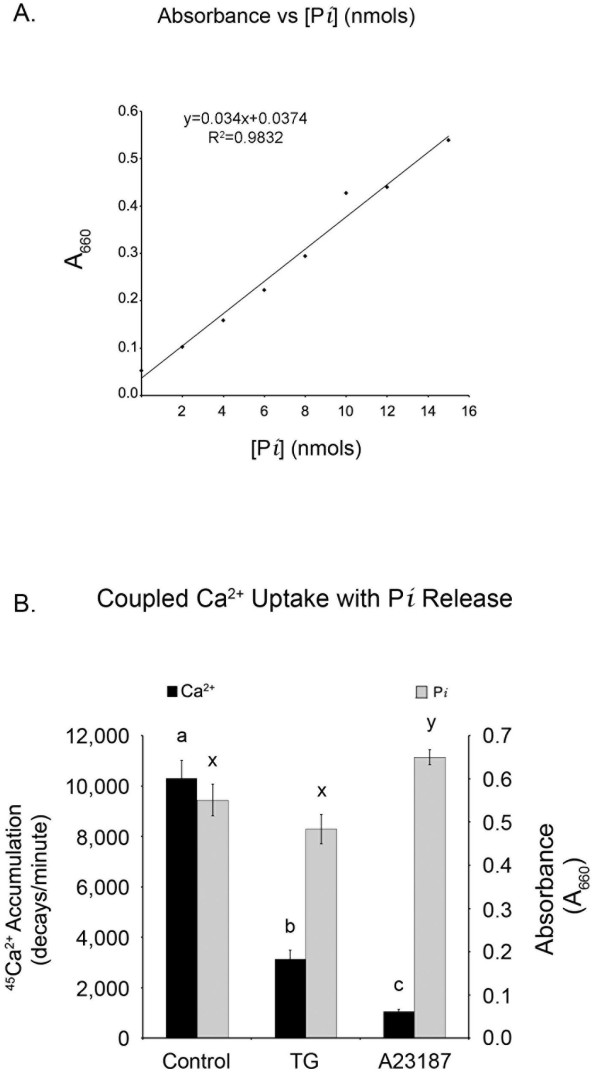
**(A) Standard curve generated using known amounts of P*i *from sodium phosphate dissolved in ultrapure water combined with the ammonium molybdate/malachite green reagent as described in materials and methods**. Color was allowed to develop for 10 minutes at room temperature before measuring A_660_. (B) Simultaneous assay measurement of ^45^Ca^2+ ^accumulation (left y-axis) and P*i *release (right y-axis) in the presence of 300 nM free Ca^2+^. Reactions were carried out with 3 mM Mg^2+^, 2 mM ATP for 10 minutes at 37°C with no additional treatments (control) or in the presence of 100 nM TG or 10 μM Ca2+ ionophore A23187.

Having optimized the assay conditions for Ca^2+ ^accumulation, the measurement of Ca^2+ ^accumulation was directly coupled to the simultaneous quantification of P*i *release (Figure 4A,B). The first step is determining P*i *concentration was the generation of a standard curve correlating absorbance at 660 nM with known concentrations of P*i *in the colorimetric reagent as shown in Figure 4A. Because there are other processes that hydrolyze ATP (both enzymatic and non-specific), P*i *released as a result of SERCA activity is defined as the difference in the presence and absence (control) of TG. Under control conditions, both Ca^2+ ^uptake and P*i *release are significantly higher (p < 0.05) than seen in the presence of TG (1 μM). The sample data collected from assays shown in Figure 4B summarizes and compares Ca^2+ ^accumulation and P*i *release in microsomes for three separate conditions: control (no additions to standard assay, see materials and methods), and addition of either 100 nM TG or 10 μM A23187. In this example, the control reaction resulted in the largest amount of ^45^Ca^2+ ^accumulated in the ER microsomal preparation as measured by liquid scintillation counting with 10,311 ± 700 counts per minute (CPM), corresponding to an activity of 7.10 nmol ^45^Ca^2+^/min/mg prot. As expected, the amount of ^45^Ca^2+ ^accumulated in ER microsomes from the same preparation containing TG, was reduced by 70% with only 3142 ± 345 CPMs (2.16 nmol ^45^Ca^2+^//min/mg protein). The difference in ^45^Ca^2+ ^uptake between control and TG represented the TG-S SERCA activity and agreed with previous reports [6,25,27]. The final treatment was the addition of Ca^2+ ^ionophore A23187. This pharmacological agent creates numerous Ca^2+ ^permeable pores in biological membranes and thus limits the accumulation of Ca^2+ ^within microsomes. Consequently net accumulation of ^45^Ca^2+ ^in the presence of A23187 was by far the lowest of the three treatment groups with only 1064 ± 74 CPMs (0.73 nmol ^45^Ca^2+^/min/mg prot). P*i *released (Figure 4B) from ATP hydrolysis was highest in the A23187 treated sample with an A_660 _of 0.650 (906 nmol/min/mg prot), followed by the control sample with an A_660 _of 0.551(756 nmol/min/mg prot), and finally the TG treated sample with an A_660 _of 0.484 (654 nmol/min/mg prot).

## Discussion

The key result of this study was the successful quantification of both Ca^2+ ^accumulation in microsomes and the release of P*i *from hydrolysis of ATP by SERCA in a 96-well format under the same experimental conditions. Calcium-mediated processes are ubiquitous throughout the cell and must be properly maintained as any significant disruption in Ca^2+ ^regulation will impair cellular function and may lead to cell death [28]. Consequently, there is keen research interest in the cellular mechanisms used to control and sequester Ca^2+ ^and use it as a second messenger. The technique described herein provides a robust tool developed from coupling two fundamental assays for investigating the effects of various metabolites, reagents, ligands, and substrates on the mechanisms of Ca^2+ ^sequestration within the ER.

The specific advantage of assaying Ca^2+ ^accumulation simultaneous with release of inorganic phosphate is a direct correlation of two linked parameters, increasing the power of data interpretation far beyond the capability from performing either assay alone. A change in ER Ca^2+ ^accumulation over time is the sum of two major processes: Ca^2+ ^accumulation, predominantly by ATP-dependent SERCA, and Ca^2+ ^release or loss through several different mechanisms, such as resident IP^3 ^and ryanodine receptor channels, or passive leak pathways. Quantification of SERCA-dependent ATP hydrolysis provides a direct measure of SERCA activity. By then comparing the net Ca^2+ ^accumulation to SERCA activity, the amount of Ca^2+ ^lost via release mechanisms and leak pathways can then be determined mathematically. This technique significantly expands upon the method introduced by Karon et al., who described a continuous spectrophotometric method to simultaneously measure changes in free Ca^2+ ^concentration and ATPase activity [29]. However, the spectrophotometric system only measured one sample at a time, which is far more time consuming, labor intensive and expensive than the technique described here, which has a capacity of analyzing 96 samples simultaneously.

The ammonium molybdate/malachite green technique of P*i *quantification is extremely sensitive, capable of reliably detecting P*i *at concentrations as low as 100 pM [22]. Due to the sensitivity of the technique, several precautions are required to ensure accurate, reliable results. We recommend running each sample in triplicate. Also, removing endogenous background levels of P*i *from a sample may be required prior to analysis if there is a low signal:noise ratio or minimal treatment effect. Endogenous P*i *is typically removed by desalting soluble protein fractions using low speed centrifugation through small columns of Sephadex G50 (300 μl of supernatant per 3 ml of G50) equilibrated in incomplete microsome homogenization buffer to remove endogenous ions, small molecules and free phosphate. Additionally, assay conditions may have to be changed depending on the specific application. For example, in Figures 1, 2 and 3, assays were incubated for 60 minutes with the goal of maximizing Ca^2+ ^uptake. In these experiments, phosphocreatine (PCr) and creatine phosphokinase (CPK) were included to regenerate ATP and maintain a constant concentration prior to experimental endpoint. Since PCr and CPK increase the P*i *background, they can be omitted for assays not requiring maximal Ca^2+ ^accumulation. Therefore, no ATP regenerating system was included in Figure 4B. Consequently, the uptake assay was incubated for only 10 minutes, to avoid depletion of ATP concentrations (2 mM).

There are many processes that may contribute to the endogenous generation of P*i*, including the plasma membrane ATPases (PMCAs), the Golgi apparatus secretory protein ATPases (SPCAs) and so on. This fact, along with exquisite assay sensitivity, helps explain why P*i *measurements do not follow strict stoichiometric rules. The fact that TG potently inhibits SERCA allows us to exploit this tool experimentally and account for these other sources of P*i*. P*i *generated from SERCA activity is defined by the difference in P*i *generated in one condition minus the P*i *generated in the same condition in the presence of TG. The concentration of TG needed to inhibit PMCA or SPCA is 10 to 300 times greater than that necessary to inhibit SERCA [30,31].

In this assay, control treatment provided maximal accumulation of ^45^Ca^2+ ^(10,311 DPMs). Maximal accumulation of Ca^2+ ^is the net difference between SERCA dependent influx and efflux through Ca^2+ ^release channels and passive leak pathways. The amount of P*i *released from ATP hydrolysis directly measures SERCA activity and was used to distinguish the relative contribution of SERCA activity compared to efflux mechanisms on the accumulation of Ca^2+^. These data represent baseline experimental conditions for comparison to subsequent treatments. As expected, the addition of TG (TG) significantly reduced microsomal Ca^2+ ^accumulation. TG irreversibly inhibits the formation of E-P intermediates of P-type ATPases such as SERCAs, thus reducing the influx of Ca^2+ ^by the preventing SERCA conformational changes and ATP hydrolysis [26]. Thus, the release of P*i *was reduced in the presence of TG (Figure 4B). In contrast, the addition of A23187, a Ca^2+ ^ionophore used experimentally to maximally induce microsomal passive leak or non-stimulated release and define non-specific activity, results in significantly reduced Ca^2+ ^accumulation within the ER microsome (Figure 4B). Despite the lowest accumulation of ER Ca^2+^, P*i *release in the A23187 treatment was the highest observed. The low levels of free Ca^2+ ^within the microsomal lumen were insufficient to inhibit SERCA activity through feedback inhibition, and consequently hydrolysis of ATP by SERCA was unabated [32].

In Figure 4B, both TG and A23187 treatments reduced the accumulation of Ca^2+^. However, without the simultaneous data demonstrating alterations in P*i *release compared to control, it would be impossible to determine if the diminished Ca^2+ ^accumulation in the presence of TG or A23187 is due to reduced SERCA activity or increased passive leak or resident release channels. These data demonstrate decreased net accumulation of Ca^2+ ^as a result of the formation of passive Ca^2+ ^leak channels in the microsomal membranes because the reduced release of P*i *indicates diminished SERCA activity. Similarly, comparison of both Ca^2+ ^accumulation and P*i *data following treatment with unknown agents will allow the attribution of alterations in Ca^2+ ^accumulation to changes in SERCA activity, release channels, or leak pathways.

In recent years, the Ca^2+^-dependence of cellular events has become even more recognized. Consequently, many new Ca^2+ ^sensors and probes have been developed to further define the role of Ca^2+ ^homeostasis in these processes. Measuring changes in cytosolic free Ca^2+ ^concentration has become quite common; however, it is now possible to measure intra- organellar Ca^2+ ^concentrations thanks to genetically encoded sensors derived from either green fluorescent protein or the jellyfish protein Aequorin [33]. These probes can be directed to the ER, Golgi, mitochondria and so on [34]. These technological marvels have both advantages and disadvantages when compared to older methods. Two major advantages include the capabilities of being targeted to individual organelles as well as being able to measure small changes in Ca^2+ ^concentration in real-time within living cells under physiological conditions. As such, the new studies will identify many potential new regulators of the Ca^2+ ^handling machinery. However, a drawback of these new techniques include the logistical difficulty of trying to elucidate the exact role of individual compounds on this machinery can be very difficult due the complex nature of live cell studies. Another limitation of these high-tech studies is that they cannot be combined with the measurement of SERCA derived P*i *production. For example, McCombs *et al.*, used a chameleon probe genetically targeted to the ER (D1ER) to measure the effect of mutations in presenilin 1 (PS1) on the concentration of free Ca^2+ ^within the ER lumen [35]. They identified mutations in PS1 that resulted in lower ER Ca^2+ ^load despite having a higher rate of ER Ca^2+ ^filling and vice versa. The only way to accurately determine the effect of the mutation of interest (or compound of interest) on the rate of SERCA is to measure the activity directly using a technique such as SERCA mediated ATPase activity and not to infer it by measuring a parameter that is the sum of many moving parts. The technique described herein will provide a valuable tool that will compliment the newer state-of-the-art Ca^2+ ^measuring techniques. Researchers aiming to quantify the effect of their compound of interest on SERCA activity and Ca^2+ ^release mechanisms can follow up their initial observations of changes in Ca^2+ ^homeostasis with quantification of the SERCA activity.

## Conclusion

The coupling of two well-established techniques to analyze mechanisms of Ca^2+ ^accumulation within microsomes provides a much more detailed understanding not only of the relative contribution of SERCA activity, but also by pharmacological manipulation and deductive analysis, the role of both active release and passive leak pathways. This technique can be easily modified to analyze not just ER microsomes, but also cell preparations and tissue homogenates, as well as a range of substrates, metabolites and pharmacological agents.

## Competing interests

The authors declare that they have no competing interests.

## Authors' contributions

DCM conducted the experiments, assisted with data interpretation, and assisted with manuscript preparation. WSK assisted with technical preparation. AV, JTC, and WDW assisted with data interpretation and manuscript preparation. All authors read and approved the final manuscript.

## Disclaimer

The opinions expressed herein belong solely to the authors. They do not nor should they be interpreted as representative of or endorsed by the Uniformed Services University of the Health Sciences, U.S. Army, U.S. Navy, Dept. of Defense or any other agency of the federal government.

## Supplementary Material

Additional file 1 **Step by step procedure**. Click here for file
