# Supplementary material for: A microplate technique to simultaneously assay calcium accumulation in endoplasmic reticulum and SERCA release of inorganic phosphate
Source: Biol Proced Online. 2012 Apr 2;14:4. doi: 10.1186/1480-9222-14-4 (PMC3388579; doi:10.1186/1480-9222-14-4)
Supplement: Additional file 1 — Step by step procedure. [file 1480-9222-14-4-S1.DOCX]

**Step by step procedure outline for figure 4**

**Isolation of microsomes.**

1. Anaesthetize rats by CO_2_ narcosis.
2. Decapitate with guillotine and remove brain over ice.
3. Homogenize whole brain (s) or brain region of interest in ice-cold homogenization buffer^1^ supplemented with 100 μM EDTA and protease inhibitor cocktail.
4. Homogenize brain tissue using motor-driven glass-teflon homogenizer.
5. Centrifuge homogenate at 10,000 x g for 15 minutes at 4°C.
6. Decant supernatant into unltracentrifuge tube and spin at 100,000 x g for 60 minutes 4°C.
7. Decant and discard the supernatant. Rinse pellet 3 times with ice-cold homogenization buffer to remove any traces of EDTA and protease inhibitor cocktail.
8. Resuspend the pellet in homogenization buffer and store on ice.
9. Quantify protein content using BCA method (or other preferred method) and dilute microsomal preparation to a final concentration of 2.5 μg/μL.
10. Aliquot microsomal preparation and store at -70°C until use.

**Preparation of inorganic phosphate colorimetric reagent.**

1. Rinse all glassware with 4M HCl
2. Prepare 100 mL of 10% (w/v) ammonium molybdate in 4M HCl. Slowly add ammonium molybdate to continuously stirring 4 M HCl to prevent clumping.
3. Prepare 300 mL of 0.2% malachite green in 4 M HCl.
4. Slowly combined the ammonium molybdate to the malachite green solution and stir at room temperature in the dark for 30 minutes.
5. Filter the colorimetric reagent through filter paper and funnel pre-rinse with 4 M HCl.
6. Discard the initial 20% of the filtrate due to dilution by the HCl rinse and store remainder in dark for up to 14 days in the dark at room temperature.

**Preparation of standard curve using known concentrations of inorganic phosphate**.

1. Prepare at 10 mM stock of NaHPO_4_ in assay buffer. Store at -20°C when not in use.
2. Dilute further and add 2, 4, 6, 8, 10, 12, and 15 nmoles of P*i* in triplicate wells in a clear 96 well microplate each in a final volume of 200 μL.
3. Add 50 μL of the colorimetric reagent per per well and allow color to develop for 10 minutes while protected from the light.
4. Measure the absorbance at 660 nM.
5. Plot A_660_ versus concentration of inorganic phosphate and calculate the equation of a straight line.

**Parallel measurement of calcium accumulation and inorganic phosphate release.**

1. Load 220 μL assay buffer^2^ per well in a 96-well 1.0 μM filter plate.
2. Load 10 μL of microsomal preparation (2.5 μg/μL)
3. Load A23187 (10 μM final concentration) in negative control wells. Radioactive counts in these wells will be subtracted out from sample wells.
4. Load 10 μL of TG into appropriate well and add 10 μL of the vehicle (DMSO) in the control wells.
5. Initiate the reaction with the addition of the pre-mix that also contains the ^45^Ca^2+^.
6. Allow the reaction to proceed for desired time at 37ºC.
7. Stop the reaction by rapid filtration and collect the eluate in clear 96 well plate.
8. Determine optimal combination of eluate, ultrapure water, and malachite green colorimetric agent.
9. Allow color to develop for 10 minutes before reading A_660_.
10. Use standard curve generated previously to convert absorbance to concentration of P*i*.

Buffers:

1. 20 mM HEPES, pH adjusted to 7.35 with KOH, 0.25 M sucrose.
2. 20 mM HEPES, pH adjusted to 7.35 with KOH, 80 mM potassium chloride (KCl), 3% (w/v) polyethylene glycol (PEG, average molecular weight of 10,000), 5 mM sodium azide (served both as an anti-microbial agent and an inhibitor of mitochondrial activity), 25 mM potassium oxalate, and 200 uM CaCl_2_ that was EGTA-chelated down to desired concentrations of free Ca^2+^.
